# Laparoscopic intercostal segment 7 resection for intrahepatic lithiasis due to low phospholipid-associated cholelithiasis syndrome. A case report

**DOI:** 10.1016/j.ijscr.2025.111490

**Published:** 2025-06-07

**Authors:** Ramiro Vargas Aignasse, Rodrigo Antonio Gasque, Germán Rodrigo Viscido, Maria Eugenia Chianalino, Micaela Noemí Ávila, Fernando Andrés Alvarez

**Affiliations:** Hepato-pancreato-biliary Surgery Section, General Surgery Department, Clínica Universitaria Reina Fabiola, Universidad Católica de Córdoba, Argentina

**Keywords:** Cholelithiasis, Hepatectomy, Laparoscopy, Phospholipids, Case report

## Abstract

**Introduction:**

Low phospholipid-associated cholelithiasis (LPAC) syndrome is a rare biliary disorder caused by mutations in the ABCB4 gene, which encodes the MDR3 phosphatidylcholine transporter. It primarily affects young adults and may persist or recur following cholecystectomy. LPAC is characterized by intrahepatic lithiasis and recurrent biliary symptoms.

**Presentation of case:**

We report the case of a 27-year-old woman with a history of recurrent acute pancreatitis and prior laparoscopic cholecystectomy, who presented with ongoing episodes of biliary colic. Laboratory tests revealed leukocytosis (12,000/μL), while liver function tests remained within normal limits. Abdominal ultrasound identified multiple echogenic foci with posterior acoustic shadowing in segment VII of the liver, along with bile duct ectasia. Further evaluation with computed tomography and magnetic resonance imaging excluded alternative diagnoses. A laparoscopic, ultrasound-guided resection of segment VII was performed. Histopathological analysis confirmed hepatolithiasis with associated acute and chronic cholangitis.

**Discussion:**

LPAC syndrome presents both diagnostic and therapeutic challenges. Although its prevalence is low—estimated at approximately 1 % among patients with recurrent biliary symptoms after cholecystectomy—early recognition is essential for appropriate management. In selected cases with localized intrahepatic lithiasis and persistent symptoms, surgical resection may offer an effective therapeutic option.

**Conclusion:**

This case highlights the importance of considering LPAC in young patients with unresolved biliary symptoms post-cholecystectomy and demonstrates the feasibility of minimally invasive liver resection in specialized hepatobiliary centers.

## Highlights

• LPAC syndrome is rare (1%), affects young women, with intrahepatic microlithiasis and biliary symptoms a rare condition with a prevalence of 1%, primarily affects young women and is characterized by intrahepatic microlithiasis and recurrent biliary symptoms following cholecystectomy
• We report a 27-year-old woman with complicated intrahepatic lithiasis, treated with lap hepatectomy.
• Lateral intercostal lap liver resection is safe and effective in experienced hands.

## 1 Introduction

Low phospholipid-associated cholelithiasis (LPAC) syndrome is a rare form of biliary lithiasis characterized by reduced phosphatidylcholine secretion into bile, resulting in increased cholesterol saturation. This supersaturation promotes the precipitation of cholesterol crystals, forming both microscopic and macroscopic stones not only in the gallbladder but also within the intrahepatic bile ducts [1,2]. The condition has a low estimated prevalence of approximately 1 % [3] and predominantly affects young women, with a female-to-male ratio of about 3:1. The average age of symptom onset is 29 years in women and 40 years in men, respectively [4].

Clinically, LPAC syndrome is characterized by recurrent episodes of biliary colic, cholangitis, or acute pancreatitis, often occurring in patients who have previously undergone cholecystectomy [5]. The diagnosis is established when at least two of the following criteria are met: (1) symptom onset before the age of 40 years, (2) recurrence of symptoms after cholecystectomy, and (3) presence of intrahepatic microlithiasis, identified on liver ultrasound as “comet-tail” artifacts, small hyperechoic foci, or biliary sludge. Although the diagnostic and therapeutic algorithms are well defined, LPAC syndrome remains underrecognized, often leading to delayed diagnosis and treatment, which in some cases requires surgical intervention [1,6].

We report the case of a young, previously cholecystectomized patient diagnosed with intrahepatic lithiasis secondary to LPAC syndrome who required surgical management. This case report has been prepared in accordance with the SCARE guidelines [7].

## 2 Presentation of case

A 27-year-old female patient with no significant medical history presented with recurrent episodes of acute pancreatitis, requiring endoscopic retrograde cholangiopancreatography (ERCP) on two occasions, the most recent occurring two months prior to consultation. On both occasions, endoscopic treatment was performed with papillotomy and successful removal of a distal common bile duct stone, without intra- or post-procedural complications. The patient was subsequently hospitalized and showed favorable clinical evolution, with no early recurrences. Her surgical history included a laparoscopic cholecystectomy performed 11 years earlier. She reported colicky abdominal pain localized to the epigastrium and right hypochondrium, without associated nausea, vomiting, or signs of cholestasis.

Laboratory evaluation revealed leukocytosis, with a white blood cell count of 12,000/μL (reference range: 4500–10,000/μL). All other laboratory parameters were within normal limits. Serological tests for viral hepatitis A, B, C, and E, as well as Epstein-Barr virus, cytomegalovirus, and human immunodeficiency virus (HIV), were negative. Immunological workup was also unremarkable, including negative results for antinuclear antibodies, anti-smooth muscle antibodies, anti-mitochondrial antibodies, anti-neutrophil cytoplasmic antibodies, anti-gp210, anti-Sp100, and normal serum IgG4 levels.

**Fig. 1:**
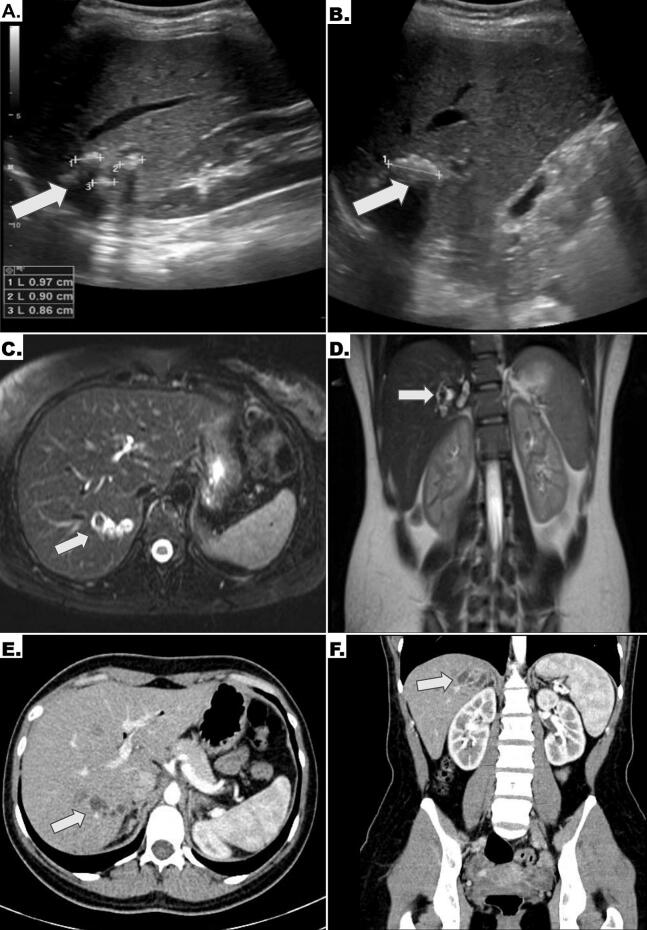
Imaging studies: Abdominal ultrasound: (A) Multiple hyperechoic foci with posterior acoustic shadowing, consistent with intrahepatic lithiasis. (B) A 3 cm hyperechoic lesion with similar characteristics located within the hepatic parenchyma. Gadolinium-enhanced magnetic resonance imaging (MRI): (C) Axial T2-weighted sequence showing hyperintense lesions associated with mild intrahepatic bile duct ectasia. (D) Corresponding coronal view of the same findings. Contrast-enhanced computed tomography (CT), arterial phase: (E) Axial and (F) coronal images showing ductal ectasia in hepatic segment VII, with no evidence of vascular malformations.

Abdominal ultrasound (Fig. 1A-B) revealed multiple echogenic images measuring between 1 and 3 cm in maximum diameter, with posterior acoustic shadowing, located in hepatic segment VII, associated with bile duct ectasia. Further evaluation with gadolinium-enhanced magnetic resonance imaging (MRI) (Fig. 1C-D) demonstrated hyperintense lesions on T2-weighted sequences without enhancement after contrast administration, consistent with intrahepatic lithiasis. Additionally, contrast-enhanced computed tomography (CT) confirmed the findings and excluded vascular malformations (Fig. 1E-F) after no suggestive signs such as anomalous vascular enhancement, arterioportal fistulas or altered perfusion patterns in the liver were observed. The absence of these findings, together with the segmental location of the lesions and their correlation with ultrasound images, allowed us to exclude this etiology.

The case was discussed in a multidisciplinary team meeting. Given the persistence of symptoms, imaging findings suggestive of complicated intrahepatic lithiasis, and the risk of future complications, surgical intervention was indicated. A laparoscopic hepatic vein-guided anatomic segment VII resection was planned.

**Fig. 2:**
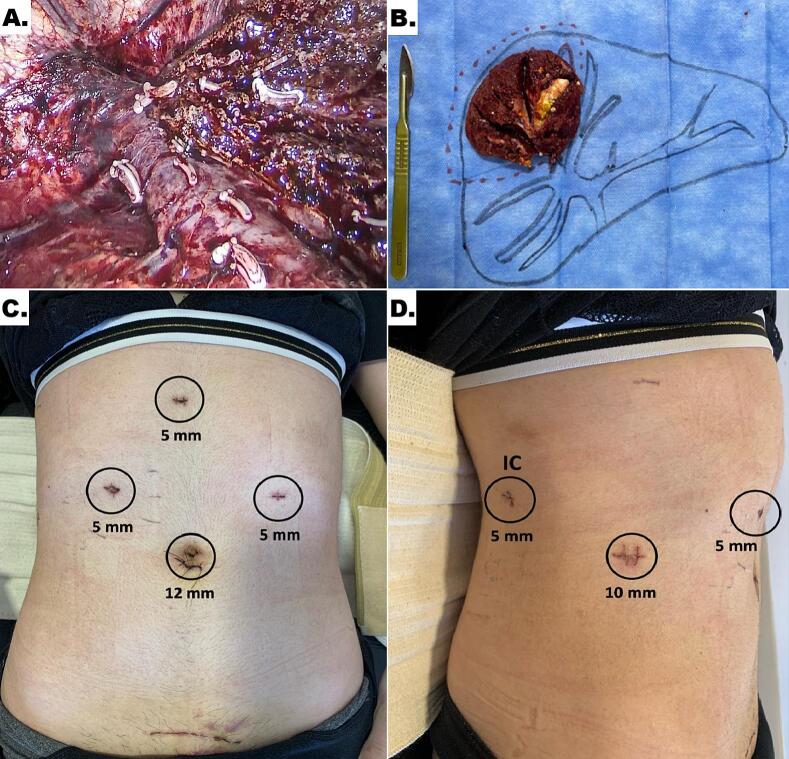
(A) Transection line after the retrieval of the specimen with the right side of the right hepatic line exposed. (B) Surgical specimen showing a large calculus associated with dilatation of intrahepatic bile ducts in segment VII. (C) Postoperative view showing the arrangement of laparoscopic working ports. IC: intercostal trocar.

A closed pneumoperitoneum was established, and six trocars were placed (Fig. 2A-B): one 12-mm trocar at the umbilical level; one 10-mm trocar in the right hypochondrium; and four 5-mm trocars located in the epigastrium, right hypochondrium, left hypochondrium, and the ninth intercostal space along the posterior axillary line. The latter was specifically positioned to facilitate right hepatic lobe mobilization and anatomic resection of segment VII.

Laparoscopic hepatectomy of segment VII was completed in a total operative time of 150 min. Five Pringle maneuvers were performed, each lasting 15 min, with 5-minute intervals of reperfusion between clampings (Fig. 2C-D). The surgical specimen was extracted via a Pfannenstiel incision and no thoracic drainage was used.

The postoperative course was uneventful, and the patient was discharged on postoperative day three. Histopathological analysis of the resected specimen confirmed the presence of hepatolithiasis associated with acute and chronic cholangitis, with no evidence of malignancy (Fig. 2E-F). At the 24-month follow-up, the patient remains asymptomatic and continues prophylactic treatment with ursodeoxycholic acid (UDCA). A follow-up magnetic resonance imaging (MRI) scan demonstrated no evidence of recurrence or residual lithiasis.

## 3 Discussion

LPAC syndrome is a rare form of biliary lithiasis, first described by Rosmorduc et al. in 2001 [2]. It is a genetic disorder caused by mutations in the ABCB4 gene (ATP-Binding Cassette Subfamily B Member 4), which encodes the MDR3 (MultiDrug Resistance 3) protein. This protein plays a key role in the excretion of phosphatidylcholine into bile; its deficiency results in cholesterol-rich, cytotoxic bile that promotes the formation of intrahepatic stones [3,4].

Although LPAC is underdiagnosed, its actual prevalence may be higher than previously thought, particularly among young women with recurrent biliary symptoms despite prior cholecystectomy. Some studies estimate that up to 25 % of women under the age of 30 who present with persistent biliary symptoms fulfill clinical diagnostic criteria for LPAC [8]. The classical diagnostic triad—symptom onset before 40 years of age, recurrence of biliary symptoms (biliary colic, jaundice, cholangitis, or pancreatitis) after cholecystectomy, and intrahepatic microlithiasis on ultrasound—is present in the majority of patients [1,3]. In our case, all major diagnostic criteria were met, reinforcing the clinical diagnosis of LPAC despite the unavailability of initial biliary lipid profiling and genetic testing. Importantly, while ABCB4 mutations are identified in only 50–65 % of patients, the diagnosis remains primarily clinical [4]. Also, it is rarely seen in adolescents and children [4,9]. Additional features that support the diagnosis include normal body weight, common bile duct stones, and absence of cholecystitis [5].

In the present case, the patient fulfilled all major diagnostic criteria: prior laparoscopic cholecystectomy, persistent biliary-type pain, prior episodes of acute pancreatitis, and ultrasonographic evidence of intrahepatic echogenic foci with posterior acoustic shadowing. Additional diagnostic modalities include bile analysis via duodenoscopy and genetic testing for ABCB4 mutations [3,8]. Bile analysis may reveal an elevated cholesterol-to-phospholipid ratio; however, this test is rarely available and requires specialized expertise. ABCB4 mutations are identified in only 50–65 % of cases [3], suggesting that genetic testing, while supportive, is not essential for diagnosis [4].

In this case, due to technical and accessibility limitations, initial biliary lipid analysis and genetic characterization could not be performed. However, the patient is currently undergoing genetic testing for mutations in the ABCB4 gene, along with members of her family. Despite these limitations, the diagnosis was made based on classical clinical criteria, which, according to current literature, is sufficient for a probable diagnosis of LPAC syndrome [4].

From a diagnostic standpoint, the role of high-quality ultrasonography cannot be overstated. Experienced operators can detect intrahepatic lithiasis with sensitivity as high as 90 %, compared to as low as 5 % among less experienced sonographers [4]. Imaging modalities such as CT and MRI are essential to rule out differential diagnoses—such as Caroli disease (absence of the central dot sign), vascular malformations or primary sclerosing cholangitis—but are less sensitive for microlithiasis [10].

In most cases, first-line treatment with ursodeoxycholic acid (UDCA) is effective (initial doses of 7–10 mg/kg/day). UDCA acts by enhancing MDR3 expression, improving phospholipid excretion, reducing bile toxicity, and promoting cholesterol solubilization [4,11]. However, as highlighted by Goubault et al. [4], a subset of patients with localized, complicated intrahepatic lithiasis—particularly when associated with segmental bile duct ectasia, recurrent cholangitis, or pancreatitis—may benefit from interventional approaches (endoscopic or radiologic). Segmental liver resection remains a valid therapeutic option in selected cases, especially when the disease is confined to a single segment and symptomatic to improve biliary drainage and ensure complete removal of intrahepatic stones [12].

Our patient presented with large stones (up to 3 cm in diameter), segmental bile duct dilation, and persistent biliary symptoms after two episodes of acute pancreatitis. These features, along with the localization of the pathology to segment VII and the absence of disease elsewhere, supported the indication for surgical intervention. Laparoscopic anatomic resection, guided by intraoperative ultrasound and performed by a specialized hepatobiliary team, allowed for definitive treatment while minimizing invasiveness [1,3].

In addition to symptom control, timely surgical management may reduce the risk of long-term complications associated with LPAC. Chronic inflammation due to retained intrahepatic stones has been associated with secondary sclerosing cholangitis, hepatic abscesses, biliary cirrhosis, and even cholangiocarcinoma in isolated reports [4,12]. Therefore, early recognition and individualized treatment plans are essential.

Despite the favorable outcome in this case, the diagnosis of LPAC remains a challenge due to limited awareness and access to specialized diagnostics such as biliary lipid analysis or genetic testing. This highlights the need for increased clinical suspicion and standardized diagnostic pathways, particularly in young patients with unexplained biliary symptoms after cholecystectomy.

Finally, further studies are warranted to better define patient subgroups that may benefit from surgical treatment and to establish standardized long-term follow-up protocols. Multicenter prospective data could help refine surgical indications and improve outcomes for patients with LPAC syndrome.

## 4 Conclusion

LPAC syndrome is a rare but clinically significant condition that requires timely and accurate diagnosis to avoid potentially severe complications. This case underscores the importance of recognizing the diagnostic criteria and considering surgical intervention as part of the therapeutic algorithm, particularly in cases of complicated intrahepatic lithiasis. Laparoscopic liver resection using the lateral intercostal approach represents a safe and effective treatment option when performed by a team experienced in minimally invasive hepatobiliary surgery.

## Author contribution

Dr. Ramiro Vargas Aignasse (writing the paper, data analysis, and interpretation, literature review).

Dra. Micaela Ávila (data analysis).

Dr. German Viscido (literature review, data analysis, and interpretation).

Dra. María Eugenia Chianalino (data analysis).

Dr. Rodrigo Antonio Gasque (revising and editing the paper, data analysis, and interpretation, literature review, study concept and approved the final version).

Dr. Fernando Andrés Alvarez (literature review, data analysis, and interpretation).

## Informed consent

Written informed consent was obtained from the patient for publication of this case report and accompanying images. A copy of the written consent is available for review by the Editor-in-Chief of this journal on request.

## Ethical approval

Given that this publication is a case report that does not contain identifiable patient information, this publication is exempt from ethical approval by the Institutional Ethics Committee of the Clinica Universitaria Reina Fabiola.

## Guarantor

Dr. Ramiro Vargas Aignasse: ramirovargasaignasse@curf.ucc.edu.ar

Dr. Rodrigo Antonio Gasque: rodrigogasque@curf.ucc.edu.ar

Dr. Fernando Andrés Alvarez: fernandoalvarez@curf.ucc.edu.ar

## Research registration number

Research registry not applicable to case report.

## Funding

No source to be stated.

## Declaration of competing interest

The authors declare that there is no conflict of interest.

## References

[1] Erlinger S. (2012). Low phospholipid-associated cholestasis and cholelithiasis. Clin. Res. Hepatol. Gastroenterol..

[2] Rosmorduc O., Hermelin B., Poupon R. (2001). MDR3 gene defect in adults with symptomatic intrahepatic and gallbladder cholesterol cholelithiasis. Gastroenterology.

[3] Rosmorduc O., Poupon R. (2007). Low phospholipid associated cholelithiasis: association with mutation in the MDR3/ABCB4 gene. Orphanet J. Rare Dis..

[4] Goubault P., Brunel T., Rode A., Bancel B., Mohkam K., Mabrut J.-Y. (2019). Low-phospholipid associated cholelithiasis (LPAC) syndrome: a synthetic review. J. Visc. Surg..

[5] Dong C., Condat B., Picon-Coste M., Chrétien Y., Potier P., Noblinski B. (2021). Low-phospholipid-associated cholelithiasis syndrome: prevalence, clinical features, and comorbidities. JHEP Rep..

[6] Belabbes F.Z., Benfaida A., Nawal B., El Idrissi Lamghari A., Rouibaa F. (2022). Low phospholipid-associated cholelithiasis: contribution of imaging in two cases. Cureus.

[7] Kerwan A., Al-Jabir A., Mathew G., Sohrabi C., Rashid R., Franchi T., Nicola M., Agha M., Agha R.A. (2025). Revised Surgical CAse REport (SCARE) guideline: an update for the age of artificial intelligence. Premier J. Sci..

[8] Condat B., Zanditenas D., Barbu V., Hauuy M.-P., Parfait B., El Naggar A. (2013). Prevalence of low phospholipid-associated cholelithiasis in young female patients. Dig. Liver Dis..

[9] Poupon R., Rosmorduc O., Boëlle P.Y., Chrétien Y., Corpechot C., Chazouillères O. (2013). Genotype-phenotype relationships in the low-phospholipid-associated cholelithiasis syndrome: a study of 156 consecutive patients. Hepatology.

[10] El Moutaoukil N., Zouaoui M., Hnach Y., Azouaoui M., Aqodad N. (2023). Considerations for low phospholipid-associated cholelithiasis (LPAC) syndrome: report of three cases. Cureus.

[11] Ikegami T., Matsuzaki Y., Fukushima S., Shoda J., Olivier J.L., Bouscarel B. (2005). Suppressive effect of ursodeoxycholic acid on type IIA phospholipase A2 expression in HepG2 cells. Hepatology.

[12] Tazuma S. (2010). The cholangiographic features of severe forms of ABCB4/MDR3 deficiency-associated cholangiopathy in adults. Gastroenterol. Clin. Biol..

